# Structural and biophysical analysis of the four CHRD domains of human chordin reveals a novel binding site for glycosaminoglycans

**DOI:** 10.1016/j.jbc.2026.113248

**Published:** 2026-06-12

**Authors:** Matthew Snee, Holly L. Birchenough, Mark H. Becker, Jonathan F. Popplewell, Hilary L. Ashe, Clair Baldock

**Affiliations:** 1 Manchester Cell-Matrix Centre, Division of Cell Matrix Biology and Regenerative Medicine, School of Biological Sciences, Faculty of Biology, Medicine and Health, University of Manchester, Manchester, UK; 2 Carterra, Salt Lake City, Utah, USA; 3 Division of Molecular and Cellular Function, School of Biological Sciences, Faculty of Biology, Medicine and Health, University of Manchester, Manchester, UK

**Keywords:** chordin, bone morphogenetic protein, BMP antagonist, heparan sulphate, X-ray crystallography

## Abstract

Chordin is a cysteine-rich protein which acts as a regulator of bone morphogenetic protein (BMP) signaling in the extracellular matrix. Acting in concert with twisted gastrulation (TWSG1), chordin works as an antagonist of BMP signaling by binding tightly to the growth factor and is a vital component of the network of interactions that establish developmental signaling gradients. Chordin is known to interact with BMP ligands *via* its four von-Willebrand factor type C domains, but the function of the large central four CHRD domains were previously unknown. Here we show that these domains interact strongly with sulfated glycosaminoglycans (GAGs) and provide evidence for the location of the binding site using X-ray crystallographic analysis combined with mutagenesis and biophysical techniques. Additionally, we report the first recombinant expression and purification of the complete functional chordin, TWSG1, BMP2, BMP7 complex which was used to demonstrate that the four CHRD domains are largely redundant with respect to the role of chordin as an inhibitor of BMP ligands. We therefore propose that the four CHRD domains of chordin have relevance in the diffusion and localization of chordin-TWSG1-BMP complexes at the tissue and organismal level, mediated by their interaction with GAGs or proteoglycans.

Bone morphogenetic protein (BMP) signaling is a ubiquitous method of cellular communication which influences a varied array of biological processes in both health and disease. Growth factors assigned as BMPs constitute 20 of the 33 known TGFβ family genes in humans (1). BMP growth factors form disulfide-linked homo- and hetero-dimers and are often glycosylated prior to secretion into the extracellular milieu. Signaling occurs *via* a hetero-tetrameric receptor complex consisting of two type-I receptors and two-type-II receptors. Upon binding of the growth factor, the type II receptors phosphorylate the type I receptor to initiate the intracellular signaling cascade. Although the expression levels of both specific growth factors and their receptors is key to the pleiotropic nature of BMP signaling, there are also a host of antagonists that influence the transport and regulation to establish productive signaling gradients.

Perhaps the best studied developmental process, in which BMP signaling is indispensable is the establishment of the dorsal–ventral axis, which has been studied extensively in both *Xenopus* and *Drosophila* model systems (2). It is now established that the appropriate signaling gradient is established by the balance of antagonists and counter antagonists, and it is notable that the system is so well conserved in Bilateria that human BMP sequences can restore normal gradient formation in *Drosophila* (3), despite the fact that the *Drosophila* gradient is inverted relative to that of vertebrates such as *Xenopus*, with high BMP signaling driving dorsalization rather than ventralization.

The most important antagonist that has been studied in this context is chordin. Initially discovered as a dorsalizing factor in *Xenopus* (4), it was found to be a central determinant in the ability of the Spemann organizer to specify the dorsal-ventral axis (5), which was already known to be reliant on a gradient of BMP4 activity. Similarly, in *Drosophila,* the chordin orthologue, Sog performs a similar function to determine the gradient of Dpp (BMP2/4) (6). The current model for chordin involves its recognition of the growth factor dimers *via* its four von-Willebrand type-C (vWC) domains (7, 8, 9, 10) and its interaction with an additional regulator TWSG1 (Tsg in *Drosophila*), which also recognizes the BMP ligand (11, 12). This cooperative mechanism whereby chordin and TWSG1 bind the growth factor and each other, produces a high-affinity complex which sequesters the ligand and antagonizes its ability to signal *via* the BMP receptors. Reversal of this antagonism is performed by the metalloproteases BMP1 and mTld (Tolloid in *Drosophila*), which cleave chordin (13) at two conserved sites (8, 14) (Fig. 1). The formation of the BMP signaling gradient therefore, requires a balance between the expression levels of chordin and TWSG1 relative to BMP1 and mTld. Chordin is known to recognize BMP2, BMP4, and BMP7 (7, 9), and there is evidence that, in *Drosophila* an interplay between Dpp (BMP2/4), and Dpp-Screw (BMP2/4 and BMP7) heterodimers might be an important factor, since *Drosophila* Sog and Tsg preferentially bind the heterodimeric growth factor (15, 16). This same work used Dpp labeling to confirm the hypothesis that the ternary complex might have a role in facilitated transport of growth factors, causing them to accumulate away from their sites of secretion (16).

**Figure 1:**
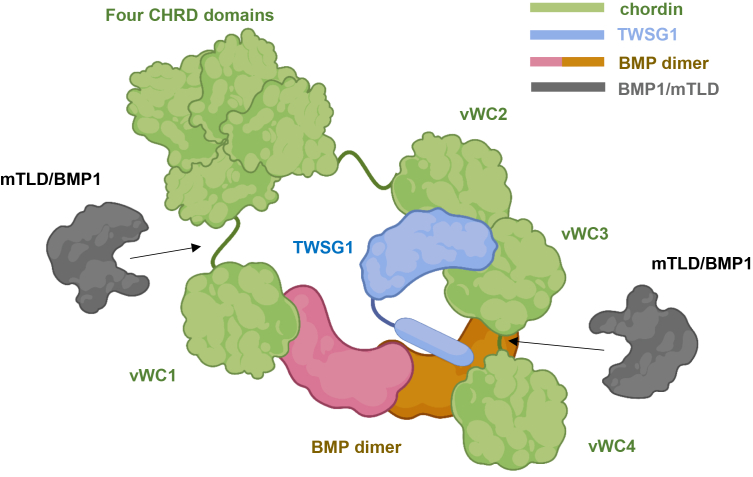
**Schematic of the chordin TWSG1 shuttling complex and its cleavage by BMP1 or mTLD.** Chordin is shown in *green*, with a BMP dimer shown in *orange* and *pink*, TWSG1 is shown in *blue* and BMP1/mTLD is shown in *black*.

Chordin itself has been studied biochemically, confirming the vWC domains as the primary sites of BMP interaction and providing evidence of the specificity of certain domains for BMP2 or BMP7 (8, 9, 10), but the large central region has not yet been assigned a function. Previous bioinformatics analysis has suggested that this region of the protein consists of four separate repeated cysteine and histidine-rich (CHRD) subdomains with a beta-barrel immunoglobulin-like fold (17). This assembly is commonly referred to as the “four CHRD region” or the “four CHRD domains” or previously as the “stem” region of chordin. The most similar proteins with known functions belong to the Cu, Zn superoxide dismutase family but it was noted that the histidine residues that coordinate the catalytic metal clusters are not conserved in chordin, and it was likely that the fold is a repurposed scaffold, utilised for a different function (17). Previous work using negative stain electron microscopy and small-angle X-ray scattering demonstrated that the subdomains form a globular assembly (8, 9), with four putative N-linked glycosylation sites (4). Evidence from *Drosophila* suggested that the glycosylation sites may have a role in moderating BMP inhibition (18).

Both chordin itself and the BMP ligands are known to interact with various components of the extracellular matrix, including glycosaminoglycans (GAGs) (19) and collagen IV (20), and these interactions are clearly an important factor in the overall regulation of the system. Biochemical studies have demonstrated affinity for heparin/heparan sulfate by the vWC domains of chordin (19), and the N-terminal regions of BMPs (21, 22, 23, 24), and links to the BMP inhibitory function have been shown (19). However, it remains unanswered why the four CHRD domains, which comprise more than half the total protein mass, are required, especially when this group of domains is absent from the related chordin-like 1 and chordin-like 2 proteins, which also function as BMP inhibitors in mammals (25, 26).

Here we describe the first experimentally-derived structures of the full assembly of four CHRD domains of human chordin, which, in combination with biophysical analysis demonstrate the presence and location of a novel GAG-binding site, and the presence of two N-linked glycans. Analysis of ternary complexes between chordin, TWSG1 and a BMP2/7 heterodimer suggests that deletion of the entire four CHRD assembly does not significantly alter the inhibitory function of the complex, indicating that its role is in interactions with glycosaminoglycans and potentially other components of the extracellular matrix. We also report the production and engineering of the full chordin-TWSG1-BMP ternary complex with implications for the *in vitro* study of BMP inhibition, and the future development of BMP-related therapeutics.

## Results

### Structure of the four CHRD domains of human chordin

**Figure 2:**
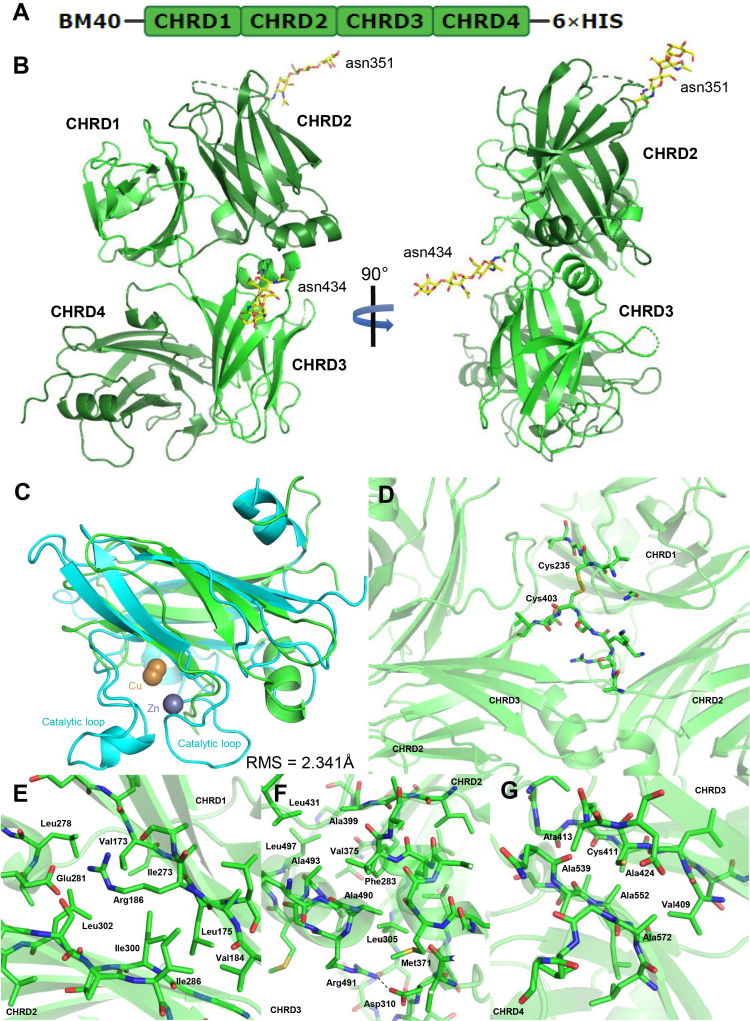
**Structure of the four CHRD domains of human chordin.***A*, schematic of the four CHRD constructs with the BM40 signal peptide and C-terminal 6 × His-tag. *B*, X-ray crystal structure of the CHRD region showing the arrangement of the four CHRD domains. The model is shown in the cartoon representation in two orthogonal orientations. Where shown, non-carbon atoms are coloured according to the CPK convention. *C*, structural alignment of the four CHRD structure (9RD6) (*green*) with the structure of extracellular superoxide dismutase (2JLP) (*cyan*). *Orange* spheres represent Cu ions, and *blue* sphere represents Zn. *D*, disulfide bonding between Cys235 and Cys403 links the domains CHRD1 to CHRDs 2 and 3. *E*, between domains CHRD1 and CHRD2, there is a hydrophobic interaction site, with an electrostatic interaction between Arg186 and Glu281. *F*, interaction between CHRD2 and CHRD3 is mediated by a hydrophobic patch and salt bridge. *G*, the interaction site between CHRD3 and CHRD4 has a minimal hydrophobic patch and features a free cysteine.

To determine the structure of the four CHRD domains, the protein was expressed in mammalian cells and purified for crystallization (Fig. S1). Several X-ray crystal structures were obtained from the multi-domain assembly, at resolutions of 2.72 Å to 3.38 Å (Table S1). The crystal structure presented here as apo (free of natural biological ligands) (PDB: 9RD6) utilized the crystallization adjuvant tellurium-centered Anderson−Evans polyoxotungstate (TEW), with chain A from this structure considered the most useful model for general analysis. The crystal structures of the four CHRD assemblies revealed four superoxide dismutase (SOD)-like domains, each with a characteristic Greek key β-barrel fold containing eight β-strands arranged in a staggered rhomboid arrangement (Fig. 2*B*). Performing structural analysis and superposition between the crystal structure of extracellular (EC) SOD (PDB: 2JLP^,^ (27)) and chain A taken from the TEW-bound four CHRD structure, resulted in best alignment with CHRD3 (root-mean-square (RMS) difference of 2.341 Å across 109 residues) (Fig. 2*C*), but the relative positioning of domains appeared largely dissimilar to the EC SOD tetramer. It is unlikely that any individual CHRD domain in chordin has SOD activity, as the extensive enzymatic loops between strands 4 to 5 and 7 to 8, which coordinate the catalytic Cu/Zn clusters in SOD1 and SOD3, are absent in chordin (Fig. 2*C*), and no metal ions were visible in these regions in the electron density maps.

A disulfide bond links residues Cys235 and Cys403 which crosslinks strand 6 of CHRD1 with the inter-domain linker between CHRDS 2 and 3 (Fig. 2*D*). All three of the four CHRD crystal structures demonstrated extensive hydrophobic interaction surfaces between each individual CHRD domain and the subsequent domain in the polypeptide chain (Fig. 2, *E*–*G*). The four CHRD domains contain a total of four potential N-glycosylation sites as predicted by sequence. Glycosylation at Asn351 and Asn434 was observed in the electron density but glycosylation at the predicted sites on Asn217 and Asn365 could not be confirmed due to the diffuseness of the density (Fig. S2).

### Flexibility and dynamics of the four CHRD domain assembly in solution

**Figure 3:**
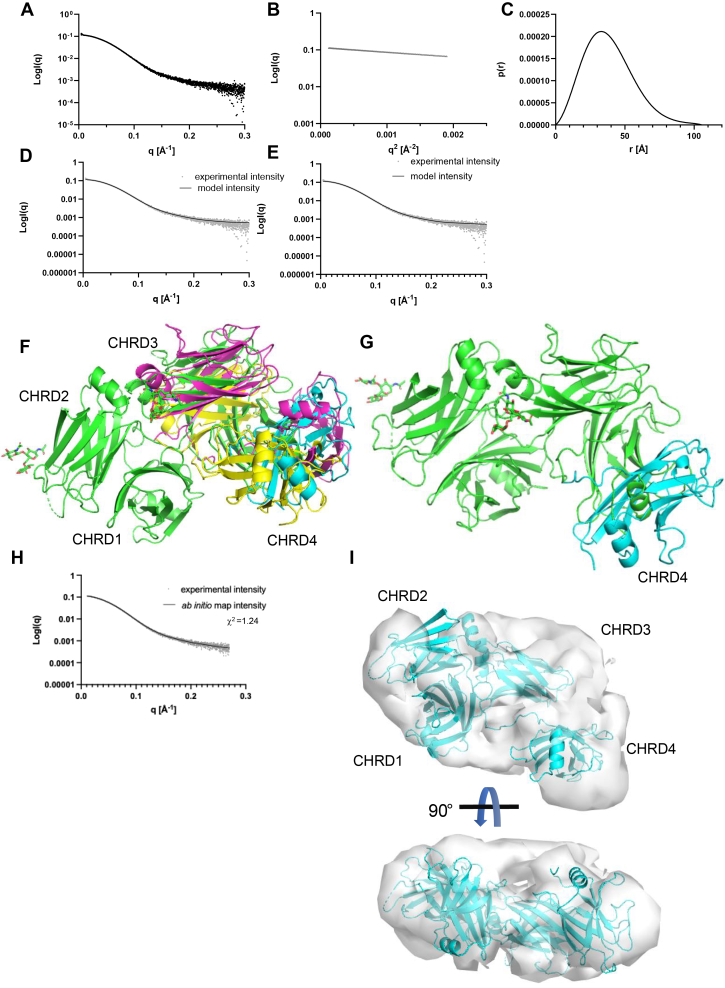
**Solution dynamics of the four CHRD domains of human chordin.***A*, experimental X-ray scattering data of the four CHRD region plotted as a function of *q*. *B*, the low *q* scattering data is represented as a Guinier plot showing the linearity of the data across the Guinier region. *C*, pair-distance distribution function *P(r)* for the four CHRD region with maximum dimension (*D*_*max*_) of 105 Å. *D*, the fit using MultiFoXS of the four CHRD region with a linker between domains CHRD3-4 which results in a single state model and (*E*) shows the fit to a two-state model resulting from linkers between domains CHRD2-3 and CHRD3-4. *F*, structural overlay of MultiFoxS derived structures, with either a flexible linker between CHRDs 3 to 4 (single state model in *blue*) or linkers between CHRDs 2 to 3 and 3 to 4 (two-state models in magenta and *yellow*), with the four CHRD structure (*green*) which were produced by fitting to the experimental solution scattering data. All models predict some movement of CHRD4. *G*, the simplest way to fit the SAXS data is *via* the movement of CHRD4 (*blue*) from the original crystal structure (*green*) *via* a single linker. *H*, the fit to the data of the *ab initio* density model generated and refined by DENSS. *I*, the *ab initio* density generated using DENSS with the multiFoxS single linker model docked using chimera.

Since the four CHRD domains of chordin form a semi-globular assembly, it appeared likely that the relative positioning of these domains would be dynamic in solution, possibly with implications for their function. Collection of small-angle X-ray scattering data on the four CHRD domains indicated that, in solution, the assembly has a maximum dimension (Dmax) of 105.7 Å and radius of gyration (Rg) of 29.6 Å (Fig. 3, *A*–*C*; Table S2). The static crystal structure fits the data poorly with a χ^2^ value of 42.03, but the fitting improves dramatically if flexibility is modelled *via* specifying mobile linkers between the CHRD domains using MultiFoXs. Flexible linkers between both CHRDs 2 to 3 and 3 to 4 as well as a single flexible linker between 3 and 4 were tested (Fig. 3, *D* and *E*). All MultiFoxs models showed some movement of CHRD4 (Fig. 3*F*), and a single linker between CHRDs 3 to 4 is the simplest way to achieve a good fit to the data (χ^2^ value of 2.28) (Fig. 3*G*). The *ab initio* density generated by DENSS also supported a model with the displacement of a single domain from the larger assembly (Fig. 3*I*). It therefore seems likely that extensive movement of CHRD4 is present in solution, with CHRDs 1 to 3 being somewhat more rigid in relation to each other.

### The role of the four CHRD domains in the inhibition of BMP signaling

**Figure 4:**
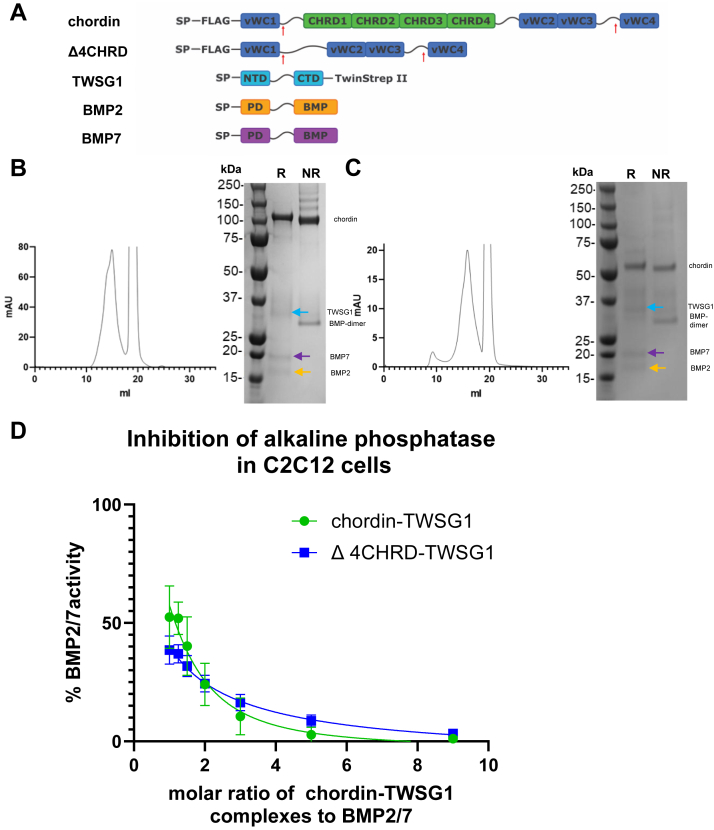
**The four CHRD domains are largely dispensable for the BMP inhibitory activity of chordin.***A*, domain schematics for the full-length chordin, Δ4CHRD variant, TWSG1, and BMP constructs with red arrows indicating mTLD/BMP1 cleavage sites. SP = signal peptide; NTD = N-terminal domain; CTD = C-terminal domain; PD = prodomain. *B*, representative size exclusion chromatography profile and reduced (R) and non-reduced (NR) SDS-PAGE for the purified full-length chordin, TWSG1, BMP2/7 shuttling complex. *C*, representative size exclusion chromatography profile and reduced (R) and non-reduced (NR) SDS-PAGE for the purified Δ4CHRD variant of chordin, TWSG1, BMP2/7 shuttling complex. In the reduced lanes shown in panels b and c, TWSG1 is indicated with a blue arrow, BMP2 is indicated by an orange arrow, BMP7 with a purple arrow. Size exclusion profiles are scaled to display protein absorbance at 280 nm with free FLAG peptide eluting at 20 ml beyond the y-axis limit. *D*, inhibition of BMP2/7-mediated alkaline phosphatase activity in C2C12 cells by full-length chordin-TWSG1 (*green*) and the Δ4CHRD-TWSG1 (*blue*). Increasing amounts of chordin-TWSG1 were titrated against chordin-TWSG1-BMP2/7 complexes to determine the IC50. 100% reflects the activity of 5 nM rBMP2/7. Data points represent the mean values from full experimental repeats (three for chordin-TWSG1 and two for Δ4CHRD –TWSG1) and error bars from standard deviation.

To probe the role of the four CHRD domains in BMP antagonism, a ternary chordin-BMP2/7-TWSG1 shuttling complex was produced *via* co-expression of chordin, TWSG1, and BMP2 and BMP7, including their prodomains, in the Expi-293F transient expression system (Fig. 4, *A* and *B*). The chordin-TWSG1 complex appeared to preferentially select the heterodimeric BMP2/7 growth factor, observed as a single band on non-reduced SDS-PAGE that resolved into two distinct monomers (Fig. 4*B*) confirmed by tryptic MS/MS as BMP2 and BMP7. In addition to full-length chordin, a ternary complex with the four CHRDs deleted (Δ4CHRD) was also generated, which removed the entire four CHRD region (Fig. 4, *A* and *C*).

To assay the ability of chordin, with and without the four CHRD domains, to antagonize BMP signaling, inhibition of alkaline phosphatase (ALP) production in C2C12 cells was measured. BMP inhibition assays typically titrate an excess of antagonist, such as chordin, against the active BMP growth factor (9, 28, 29). As the ternary chordin-TWSG1-BMP2/7 complex contains both the antagonist and growth factor, increasing concentrations of chordin-TWSG1 or chordin Δ4CHRD-TWSG1 complexes that did not contain the BMP growth factor were combined with a fixed concentration of the respective ternary complex to generate an excess of the antagonist. To determine the percentage of BMP inhibition, the inhibition of ALP was normalized to the activity of BMP2/7 alone (positive control). These results show that, in the presence of TWSG1, both full-length chordin and Δ4CHRD inhibit ALP induction similarly (Fig. 4*D*). Analysis of the inhibition using nonlinear fit with a variable slope determined an IC50 of 6 nM (molar ratio 1.205:1) for full-length chordin-TWSG1 and 4 nM (molar ratio 0.897:1) for Δ4CHRD-TWSG1. However, as the putative IC50 value for the Δ4CHRD complex lies outside of the tested range of molar ratios (which starts at 1:1 for the ternary complex) and due to the overlapping standard deviations, these differences were not meaningful. Therefore, it appears that the four CHRD domains are largely dispensable for the inhibition of BMP signaling by chordin.

### The role of the four CHRD domains in cleavage of chordin complexes

To determine whether BMP1 cleavage of the ternary complex is influenced by the four CHRD region, the rate of cleavage of the full-length chordin and Δ4CHRD complexes were tested (Fig. S3, *A*–*C*). These data show that both the full-length and Δ4CHRD complexes undergo rapid initial cleavage but cleavage at the N-terminal site downstream of vWC1 is absent in the Δ4CHRD complex (Figs. S3 and S4). Further discussion of these results is included in the supporting information.

### The four CHRD domains as a site for interaction with glycosaminoglycans

**Figure 5:**
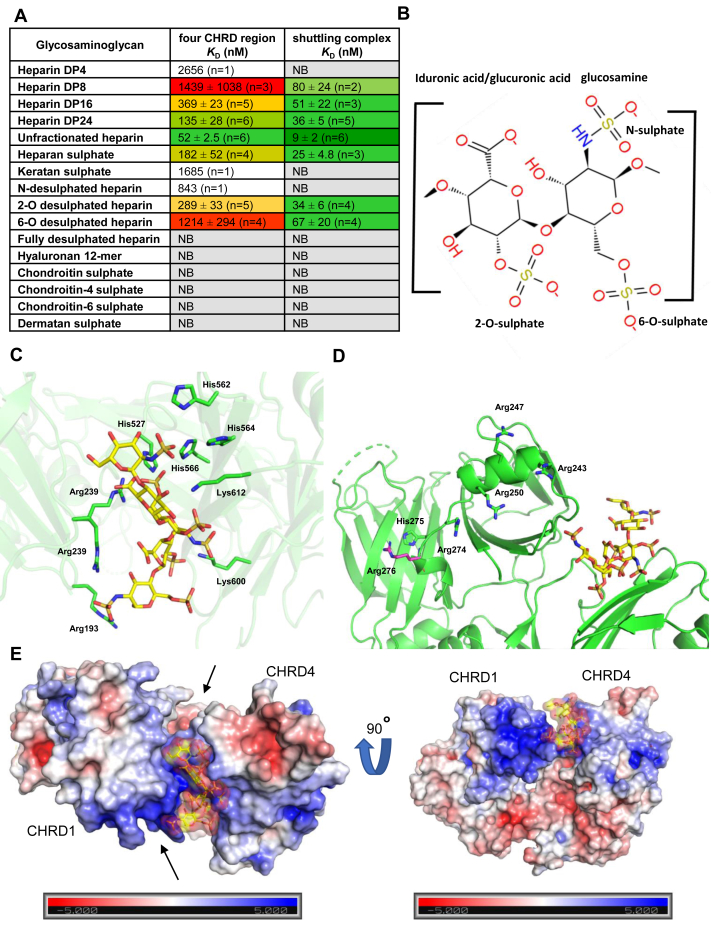
**Glycosaminoglycan binding site in four CHRD domains of human chordin.***A*, binding to a range of GAGs was screened by SPR using a high throughput GAG array on the Carterra LSA^XT^. Equilibrium dissociation constants for the four CHRD region and the full-length chordin-TWSG1-BMP2/BMP7 shuttling complex were determined. Affinity values are colour coded from green (strongest binding) to red (weaker binding). *Gray* cells indicate samples where no binding (NB) was detected, white cells indicate samples where weak binding was observed in only one replicate. Number of replicates is indicated (N = 1–6). *B*, diagram of a canonical heparin disaccharide indicating 2-O and 6-O sulfation positions. *C*, structure of the CHRD region bound to heparin (shown in *yellow*). Positively charged residues that interact with sulfate groups in the GAG binding site between CHRDs 1 and 4 are shown. *D*, additional basic patch located on one side of CHRD1 with a putative disease associated variant at Arg276 (Arg276Gln) (shown in *pink*). *E*, electrostatic surface of the four CHRD domains shown in two orthogonal orientations. Arrows indicate an extended positively charged patch on CHRD1.

Since the four CHRD domains did not appear to be directly involved in the BMP2/7 antagonism, we tested whether it shared the glycosaminoglycan-binding properties of extracellular SOD (30). Using a high throughput GAG array, it was shown that the four CHRD domains and the chordin-TWSG1-BMP2/7 ternary complex do indeed interact strongly with sulfated heparin, heparinase-derived oligomers, DP8, DP16, DP24, and heparan sulfate (Figs. 5*A* and S5). Neither the four CHRD domains or ternary complex interacted strongly with chondroitin or dermatan sulfate, and the affinity was lost when the sulfate groups were removed from heparin. These data indicate that the isolated four CHRD domains and the larger complex both interact with sulfated glycosaminoglycans *via* their sulfate groups. Additionally, the higher density of sulfation seen in heparin and heparan sulfate compared with dermatan or keratan sulfate is a requirement for strong binding, suggesting that a relatively large number of simultaneous charge-based interactions occur between the CHRD domains and a sulfated glycosaminoglycan chain.

### Mapping the glycosaminoglycan binding site

To confirm binding with heparan sulfate (HS) and map the position of the binding site in the larger assembly, a structure of a four CHRD-HS complex was determined at 2.92 Å. Combined co-crystallization and soaking with short heparin oligomers produced a crystal structure where the ligand could be recognized binding a highly basic region between CHRD1 and CHRD4 with five heparin monomers resolvable (Fig. 5, *C*–*E*). The heparin oligomers used are naturally heterogeneous in their composition, but the position of sulfates interacting with clusters of positively charged sidechains was clear (Fig. S6), and the position of the linkages between monomers made it possible to determine the direction of the binding. The first cluster of basic residues consisted of Arg193, Arg239, and Lys600, with the second involving His562, His564, His566, and Lys612 (Fig. 5*C*). His527 probably also contributes to interactions with sulfated GAGs but is poorly resolved so its involvement must be considered tentative. An additional patch of net positive charge exists on the surface of CHRD1 (Fig. 5*E*) which features a putative pathogenic variant Arg276Gln (31). This region could also be involved in the binding of larger GAGs. In the crystal, the putative GAG binding site faces the equivalent site of a symmetry mate (Fig. S6, *A* and *B*), and the precise binding pose involves interactions between the ligand and Arg597, Arg239, His564 and possibly Lys612 from this adjacent chain. This constraint also appears to limit the size of the GAG that can bind and explains why useful diffraction from crystals containing DP8 or any longer oligomer could not be obtained. In summary, the heparin bound crystal structure appears informative as to the general position of the site between CHRDs 1 and 4, and the regions which recognise sulfate residues in the natural ligands, but specific inferences about the binding mode of larger GAGs in solution should be made cautiously.

### Studying the effect of mutations and four CHRD deletion on the interaction with glycosaminoglycans

**Figure 6:**
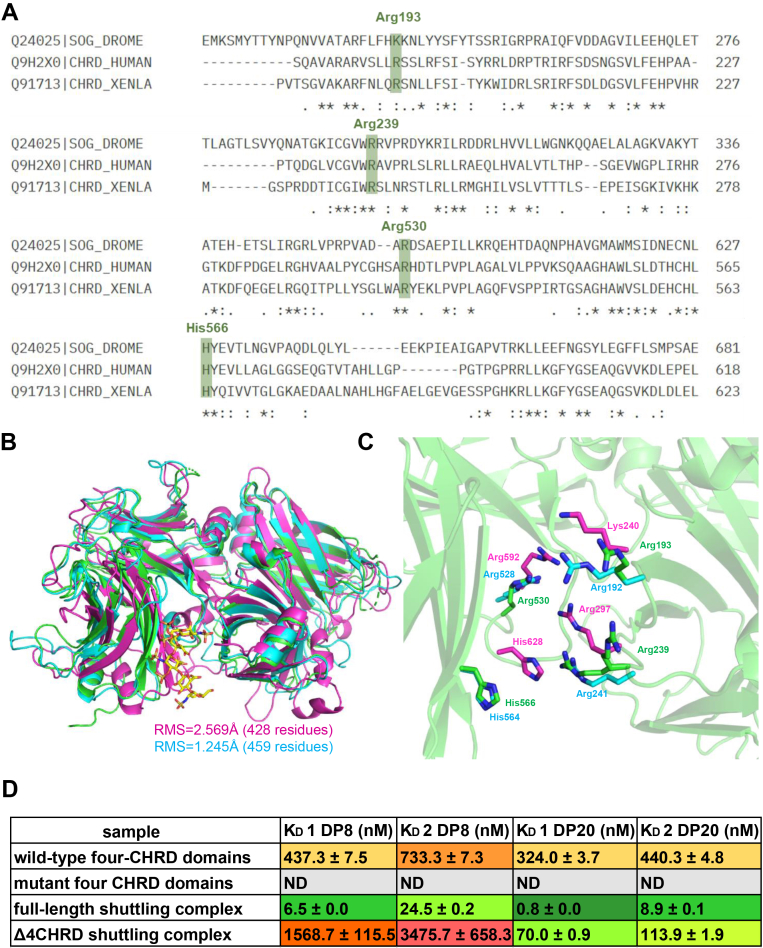
**Disruption of glycosaminoglycan binding in the four CHRD domains and shuttling complexes.***A*, multiple sequence alignment of domains CHRD1 and CHRD4 between human chordin, *Drosophila* sog, and *Xenopus* chordin. Green indicates sites where a residue with net positive charge is conserved between all three organisms at this position. *B*, structural alignment of the four CHRD domains (heparin bound form in *green*) to the Alphafold-3 predicted region from *Drosophila* Sog (*magenta*), and *Xenopus* chordin (*blue*). The root mean square deviation (RMS) between C-alpha atoms is indicated. *C*, structural conservation of the binding site in the four CHRD region between human chordin (*green*), *Drosophila* sog (*magenta*) and *Xenopus* chordin (*blue*). *D*, dissociation constants for wild-type and mutant four CHRD region, full-length shuttling complex, and Δ4CHRD complex, binding to DP8 and DP20 determined by Octet-BLI. Data were fitted to the heterogeneous ligand model, producing two K_D_ values. Affinity values are colour-coded from *green* (strongest binding) to red (weakest binding) to indicate relative binding strength. ND indicates not determined, as the amount of mutant protein binding to HS was dramatically reduced compared to wild type.

To probe the biological relevance of the identified HS-binding site, the structural and sequence conservation of the binding site was compared in the four CHRD region of the model organisms, *Drosophila* and *Xenopus*, which have conserved BMP signaling pathways. AlphaFold3 (32) predictions of the four CHRD region in the *Drosophila melanogaster* orthologue Sog (pTM = 0.92) and chordin from *Xenopus laevis* (pTM = 0.9) were generated, and structural and sequence alignment was performed to identify conserved basic residues that were likely to interact with the sulfates from GAGs (Fig. 6, *A*–*C*). This resulted in the identification of R193, R239, R530, and H566 in chordin which correspond to K240/K241, R297/R298, R592, and H628 in Sog, and were absolutely conserved between human and *Xenopus laevis* (Fig. 6, *A* and *C*). In order to perturb the HS binding site, residues R193, R239, R530, and H566 in the four CHRD construct were mutated to alanine.

To assess the stability of the mutant protein in solution, static light scattering measured from 20 to 90°C was used to determine aggregation temperatures of 53.1 °C and 50.1 °C for the wild-type and mutant CHRD proteins (Fig. S7). These data indicate that the R193A/R239A/R530A/H566A mutant four CHRD region is stable in solution with a small decrease in aggregation temperature reflecting the change in surface entropy and increase in hydrophobicity resulting from the mutation of four basic residues to alanine. The R193A/R239A/R530A/H566A mutant four CHRD protein was therefore used in crystallization trials, and a crystal structure was obtained from an I222 crystal form, which at 2.72 Å has the highest data resolution. Where the decrease in surface entropy likely allowed this crystal form, and although the optical resolution is increased, the B-factors indicate there is still flexibility between the CHRD domains. Taken together, the light scattering and structural data indicate that the mutations do not disrupt the fold of the human four CHRD domains or their assembly.

To gain affinities for the binding of the R193A/R239A/R530A/H566A mutant and wild type four CHRD domains to HS, as well as the full ternary shuttling complex and Δ4CHRD shuttling complex, these samples were analyzed against heparin oligomers using the Octet BLI platform (Fig. S8). Data were analyzed using the heterogeneous ligand model to account for the heterogeneity in heparin sulfation patterns which yielded two K_D_ values (Fig. 6*D*). These data indicate that the wild-type four CHRD domains bind strongly to both DP8 (K_D_1 437.3 nM, and K_D_2 733.3 nM) and DP20 (K_D_1 324.0 nM, K_D_2 440.3 nM). However, the response from the mutant protein was dramatically reduced so the affinity was not determined (Figs. 6*D* and S8*A*), suggesting that the mutations were successful in disrupting the binding of sulfated GAGs. The full shuttling complex containing chordin, TWSG1, and a BMP2/BMP7 heterodimer was found to have a very high affinity for DP8 (K_D_1 6.5 nM, K_D_2, 24.5 nM) and bound DP20 with even higher affinity (K_D_1 0.8 nM, K_D_2, 8.9 nM) representing the additional contribution of the HS-binding sites on the vWC domains and BMP ligand (19, 21, 24, 33). Deletion of the four CHRD domains in the shuttling complex (Δ4CHRD) resulted in a large reduction in the affinity for DP8 (K_D_1 1568.7 nM, K_D_2 3475.7 nM) and DP20 (K_D_1 70.0 nM, K_D_2 113.9 nM) (Figs. 6*D* and S8). These data reveal that a great deal of the overall affinity of chordin for GAGs resides within the four CHRD domains, but the combined contribution of the sites on the vWC domains and BMP ligand are still significant. As heparin and heparan sulfate enhances collagen cleavage by BMP1 in the presence of the enhancer PCPE1 (34), cleavage of the chordin complexes by BMP1 was also performed in the presence of unfractionated heparin and DP20 (Fig. S3*E*). However, there was no change in the rate of cleavage of chordin by BMP1 indicating that cleavage of chordin is not enhanced by HS.

## Discussion

Here, we describe the experimentally derived structures of the four CHRD domains from human chordin and show that this region interacts with glycosaminoglycans at a site between CHRD1 and CHRD4. It has long been known that BMPs and other TGFβ family members exhibit strong affinity for GAGs (24, 33, 35, 36, 37), as do many of their inhibitors including gremlin (38), BMPer (39), Noggin (40), and chordin (19).

It has been previously demonstrated that the binding of chordin to the cell surface occurs *via* interaction with heparan sulfate proteoglycans (HSPGs) such as syndecan-1 and syndecan-4, and that these interactions, which occur *via* the sulfate moieties on the GAGs, are important for the inhibitory activity of chordin against BMP4. The same work mapped these binding events to the vWC domains but did not analyze the contribution of the additional domains (19). Interestingly, work exploring the effect of HSPGs with relation to fibrodysplasia ossificans progressiva, showed that removal of GAG chains using heparinise in wild-type cells reduced the transcription of the BMP reporter ID1 in response to BMP4 (41), and exogenous heparin has also been shown to inhibit BMP4 signaling, presumably by blocking interactions with cell-surface HSPGs (42). It seems likely that, in the absence of any exogenous inhibitors, cell surface proteoglycans may promote BMP signalling by localizing the ligands to the cell surface, but they can conversely act as the nexus for the formation of inhibitory complexes depending on the availability of proteins such as chordin and TWSG1 in the local environment.

It is less clear why chordin features such an abundance of GAG binding sites. Between the four vWC domains, the BMP dimer, and the additional site described here, it is possible that there are as many as seven individual regions that recognize sulfated GAGs in the complete ternary complex. This suggests that there could be a biological requirement for the extremely high affinity between the complexes and GAGs which was observed in this work. Cooperativity between multiple sites may be required to compete with the BMP receptor, since growth factor-receptor interactions can feature K_D_ value in the low picomolar range such as the 26 pM value observed for AKL3/BMPR1 and BMP2 (43). A second possibility is that GAG binding is still relevant in chordin and its complexes once they have been partially or fully cleaved by tolloid/BMP1 proteases (8). Removing the four CHRD domains from the complex appears to have a greater impact on binding when the GAG has a shorter chain length, with a reduction in K_D_1 affinity of 241-fold for DP8, compared to an 88-fold change for DP20 (Fig. 6*D*). The affinity of all samples for DP20 is higher than for DP8, so it is plausible that fewer sites can engage simultaneously when the GAG is shorter. The differences between the K_D_ values determined from the Carterra LSA^XT^ SPR and Octet BLI data, may reflect the lack of a heterogenous ligand analysis model for the Carterra system. Although some of the Octet BLI K_D_1 values indicate very tight interactions between a subset of ligands (presumably with the most favorable sulfation pattern), these may not necessarily be the most abundant species in the sample and suggests that the exact composition of the GAG may be a strong determinant of its affinity. The K_D_ values from the Carterra LSA^XT^ for heparin DP8 show broad agreement with the K_D_2 values from the Octet, which may indicate that the majority of species in the naturally heterogeneous preparations bind with affinities similar to those observed in the Carterra LSA^XT^ experiment. However, certain specific sulfation patterns may bind more tightly reflecting the K_D_1 values determined in the Octet experiment.

Whatever the relevance of the additional GAG binding site identified between the CHRD domains in this work, it is still not obvious why a large multi-domain assembly is preserved in chordin when the smaller vWC modules are also capable of recognizing GAGs. It seems somewhat likely that the four CHRD domains have additional functions including protein-protein interactions with extracellular matrix proteins. The effect of removing the four CHRD domains from chordin on the abundance of the N-terminal cleavage fragment may indicate some interaction with BMP1. It is also possible that the four CHRD region serves as an interaction site for a protease enhancer, although it is notable that the known BMP1 enhancer PCPE1 has been tested against chordin without displaying any potentiating effect (44). Other possibilities for protein interaction partners have yet to be explored. In work on *Xenopus*, chordin was found to diffuse in a region rich in extracellular matrix including fibronectin (45) so putative interactions with various fibrillar matrix proteins may be worthy of study.

### Production of chordin-TWSG1-BMP ternary complexes

This work represents the first report of a recombinant shuttling complex containing chordin, TWSG1 and a BMP growth factor dimer. Whilst growth factors have been previously refolded from bacterial sources or isolated from mammalian culture and reconstituted into complexes with inhibitors for structural and functional analysis (10, 11, 29), here it was possible to co-express all the proteins and purify a complex by sequential affinity steps and size exclusion, sidestepping problems with insolubility, which are commonly experienced with the mature growth factors. The apparent lack of BMP2 and BMP7 homodimers under oxidizing conditions (Fig. 4, *B* and *C*) suggests that the BMP-binding domains of chordin and TWSG1 have a significant preference, and by extension, an increased affinity for heterodimeric growth factors relative to homodimers. This observation is in keeping with the model proposed for the *Drosophila* proteins (16), but since experiments to confirm the relative abundance of homo and heterodimers in the expression system, their relative solubility, and behavior in non-reducing SDS PAGE are beyond the scope of this work so this observation should be considered as an area for future study.

Although the focus of this work is on the structure and function of the four CHRD domains, the exploration of internal truncation of this domain and the assays that show BMP target gene induction at varying molar ratios, give some sense of the potential for exploring the function and specificity of chordin and TWSG1 both *in vitro*, and in cell or even animal models. Since they can stabilize and solubilize active growth factors in standard aqueous buffers which can be challenging (46) it is also possible that these complexes could serve as a starting point for new BMP-based or BMP inhibitory bio-therapeutics and offer alternatives to the carriers often employed for BMP treatments (47, 48, 49, 50). Use of a recombinant natural inhibitor as the solubilizing partner could offer the potential to modulate BMP release in a tissue specific manner and suggests the possibility of antibody-fusion approaches to target BMP ligands to certain cell types.

In summary, this work demonstrates experimentally that the four CHRD regions in human chordin does not appear necessary for BMP binding and inhibition, but instead binds sulfated GAGs, primarily *via* charge-based and polar interactions with their sulfate groups at a site between CHRD1 and CHRD4. Additionally, the methods described here for obtaining recombinant samples of full ternary shuttling complexes will be valuable for advancing the understanding of BMP recognition and inhibition by chordin and TWSG1 and may have scope to be extended to other growth factor–regulator complexes.

## Experimental procedures

### Expression constructs

Full-length human chordin was expressed from a plasmid which encoded residues 27 to 955 with an N terminal FLAG tag downstream of the signal sequence from receptor-type tyrosine-protein phosphatase S. This sequence was cloned into pHTBV1.1-CT-GFP which was a gift from the Centre for Medicines Development (CMD) in Oxford and subject to a MTA for academic use with the creator of the original Bac-mam system, Frederick M. Boyce (51). This construct is competent for the creation of Bac-mam viruses for large-scale protein production, but this was not utilised in this case. The four CHRD deletion construct was derived from the full-length construct, but residues 168 to 657 were replaced with the linker SASAT and is hereafter referred to as Δ4CHRD or Δ4CHRD complex, where it is bound to TWSG1 and/or BMP2/7. The wild-type four CHRD construct has been previously described (9), and encoded residues 168 to 652 of human chordin downstream of the BM40 (SPARC) secretory signal peptide. This construct also contained a C-terminal thrombin-cleavable His-tag and was cloned into the pCepPu/-Ac7 vector. The mutant four CHRD construct was similar in all regards aside from the mutations, the use of the pCep-Hyg plasmid in place of the puromycin resistance version, and additional codon optimization during the synthesis of the mutant sequence. The four CHRD-only constructs contain a natural variant M630L, which was preserved between the wild-type and mutant four CHRD constructs, for direct biophysical comparison, but is not present in the full-length construct. The construct encoding TWSG1 contained residues 26 to 223 cloned into phLSEC (52) using the included receptor-type tyrosine-protein phosphatase S signal sequence and a C-terminal Twin-strep tag. The BMP2/BMP7 heterodimer was produced from the pVAX1-BMP2/7+ vector created by Georg Feichtinger (53) and obtained through Addgene.

### Protein expression and purification

All proteins used in this study were expressed using the Expi293F transient expression platform (Thermo) and were expressed according to the manufacturer’s instructions. Conditioned media was passed over a packed column of affinity resin using gravity flow. Shuttling complexes (containing N-terminal FLAG-tagged chordin, and C-terminal strep-tagged TWSG1) were purified using strep-tactin XT superflow with Biotin-block added to the media prior to application to the resin. The strep-tactin resin was washed with 25 mM HEPES pH 7.5, 150 mM NaCl (buffer A) and then eluted directly onto a FLAG column using the same buffer with the addition of 50 to 70 mM Biotin. The FLAG column was then washed with buffer A and the protein was eluted using 500 μg/ml 3X FLAG peptide. This elution was immediately injected onto a Superose 6 Increase 10/300 Gl column (Cytiva) for gel filtration chromatography in buffer A. Peak fractions were pooled and analysed by SDS-PAGE to confirm the presence of the correct species (Fig. S1). His-tagged proteins such as the four CHRD constructs were purified in a broadly similar way, except with Ni EXCEL resin (Cytiva) and the addition of 5 mM imidazole to the media prior to purification. The resin was washed with buffer A with the addition of a 35 mM imidazole followed by elution with 300 mM imidazole. Concentrations of proteins were estimated using a nanodrop spectrophotometer, although the theoretical extinction coefficients which were generated by PROTPARAM (54) were validated using the BCA technique using a micro-BCA kit (Thermo) and found to be accurate, and SDS PAGE was also used to confirm that samples at the same estimated concentration gave similar band intensity, most importantly the BMP band from the shuttling complex variants, and the wild-type and mutant four CHRD region (Fig. S1). When not required immediately, proteins were flash frozen in liquid nitrogen and stored at −80 °C.

### Crystallization and structure solution

Crystallization of the four CHRD domains expressed from the pCEP C-terminal His-tagged construct was accomplished at 8 to 12 mg/ml using the *in situ* proteolysis technique. Bovine trypsin type XI was applied prior to concentration to limit the contamination of the final sample with salts or other small molecules. Trypsin was dissolved in buffer A and was added so that the final concentration would reach approximately 0.05 mg/ml once the Chordin sample had reached its desired concentration. Crystallization trials were performed using the sitting drop technique using SwissCi Plates and mosquito pipetting robots (TTP labtech) at the MPSF (Manchester protein structure facility). Hits of varying quality were recorded in several commercial screens including Morpheus, PACT, and the LMB screen (Molecular dimensions). The Anderson−Evans polyoxotungstate (TEW) bound form of the four CHRD domains was obtained from digestion of the entire BMP2/BMP7-bound shuttling complex which was trialed at 3 mg/ml with 10 mM TEW.

Crystals were harvested and frozen in liquid nitrogen for data collection using the automated UDC pipeline and Diamond Light Source in Oxfordshire (proposal MX31850) at beamlines I04 and I03. Data was integrated using Xia2 Dials (55, 56) and scaling and merging was performed using Aimless (57). For the heparin-bound structure, two integration outputs were identified by BLEND (58) and combined at the scaling step. Structure solution was performed by molecular replacement using search models from the Alpha Fold DB (32, 59), followed by rebuilding in COOT (60, 61) and refinement using Phenix.refine using automatic Ramachandran restraints (62) and ligand restraints generated using ACEdrg (63). Validation was performed using Molprobity (64). Details of data quality and refinement can be found in Table S1. Crystal forms appeared atypical with a high degree of conformational flexibility relative to data resolution, and high solvent content (more than 75% for the heparin-bound form), with the Wilson-B factors and individual ADPs strongly affected by the conformational flexibility. Attempts to resolve this and recover appropriate B-factor values for individual atoms were unsuccessful. Since these data may benefit from future improvements in analysis software, the raw images were uploaded to Zenodo.

### Cell signaling assays

Assays studying the induction of alkaline phosphatase in mouse C2C12 myoblast cells were performed in DMEM media with high glucose and the addition of 2% non-essential amino acids (Sigma), 2% glutamine (Sigma), and 1% Penicillin streptomycin (Thermo). Growth media was supplemented with 10% FBS, whereas assay media contained 0.1% FBS. Cells were seeded in clear-bottomed black tissue-culture treated CellStar plates (Greiner) at a density of 10,000 cells per well in 150 μl and grown for 24 h prior to treatment. Test samples were prepared using serial dilutions at 10X the final concentration prepared in assay medium. Growth media was removed from cells and replaced with 135 μl of assay media and 15 μl of treatment and cells were incubated for 24 h. Plates were then prepared by the removal of the media, washed with Dulbecco’s PBS and the addition of 100 μl of lysis buffer containing 50 mM Tris HCL pH 9.5, 150 mM NaCl, 0.5 mM MgCl_2_, 1% v/v Triton X-100, and 1 mM 4-Methylumbelliferyl Phosphate. Plates were then incubated at room-temperature for 1 h prior to reading on a fluorescence microplate reader. Inhibition assays were normalized to a growth factor-free control and then expressed as a percentage response relative to 5 nM commercially available recombinant free BMP2/7 growth factor (R&D systems). Data were analyzed using Graphpad PRISM with a variable slope and assumed a 1:1 ratio of chordin to BMP dimer in the ternary complexes. The “top” for curve fitting was constrained at 100% activity to reflect the assumption that the unmeasured 0:1 M ratio would produce a similar signal to the rBMP2/7 normalization control (0% inhibition at a 0:1 M ratio of chordin-TWSG1:BMP2/7). Experiments were performed with three full experimental replicates (separate protein preparations and cell passage) for the full-length, and two full experimental replicates for the Δ4CHRD complex. Each experimental replicate contained at least eight technical replicates with a total N = 56 for the full length and N = 72 for Δ4CHRD were weighted equally.

### Chordin cleavage assays

Cleavage of chordin complexes was performed using recombinant BMP1 (rBMP1) (R&D) in 25 mM HEPES pH 7.5, 150 mM NaCl, with 5 mM CaCl_2_. Reactions were performed at 100 μl scale with 100 ng rBMP1 (approx. 14 nM). Each digestion of a full-length complex contained 18 μg of complex, whereas the Δ4CHRD reactions contained 12 μg of complex to maintain the same molarity (1.15 μM) and not alter the kinetics of cleavage. Heparin-containing reactions contained 5 μg of heparin. Assays were run at 37 °C with 15 μl samples taken at 0, 30, 60, 120, and 240 min, boiled in reducing SDS PAGE loading dye and frozen. Samples were analyzed using Coomassie-stained SDS PAGE. Gel densitometry was performed using Image Lab software (Biorad) using the volume integration tool with background subtraction. Integrated values for chordin bands were normalized against background and expressed as a proportion of the value for the no-enzyme control which was run as a separate incubation. These values were multiplied by 100 and subtracted from 100 to produce values for the percentage of chordin in each complex that has undergone at least one cleavage event. Experiments were performed in triplicate as a minimum. For Western blot detection of reaction products, a rabbit anti-FLAG (cell signalling 14793S) primary was used in combination with an alexa 647 conjugated donkey anti-rabbit secondary. Gels and blots were imaged using a Bio-Rad Chemi-doc system.

### Biological small-angle X-ray scattering (BioSAXS)

BioSAXS data were collected on beamline B21 at Diamond Light Source (65). The energy was 13.1 keV with a wavelength of 0.9464 Å, a photon flux of 4 × 10^12^ s^−1^, and a recorded q-range of 0.0045 to 0.34 Å^−1^. Samples were loaded onto a Superdex 200 Increase 3.2/300 column, pre-equilibrated in HBS and running at 0.075 ml min^−1^ for inline SEC-SAXS. Eluted samples passed through the beam and 1.0 s exposures were collected on an EigerX 4M detector (*Dectris*). Data were normalized against the primary beam intensity, and absolute intensity was scaled to water (0.0163 cm^−1^) using Diamond’s Data Analysis WorkbeNch (DAWN) (66). Data were merged, buffer subtracted to produce 1d line profiles and analyzed in ScÅtter IV. Experimental scattering data were compared to experimental atomic models using FoXS, but these structures had a relatively poor fit to the experimental SAXS data. MultiFoXS (67) was used to sequentially introduce flexible linkers between CHRD domains to allow movement of individual or all CHRD domains. The models with best fit to the scattering data involved the movement of CHRD4. *Ab initio* density with a resolution estimate of 36 Å was generated from the scattering data using DENSS (68) and multiFoxS models were docked using Chimera (69).

### High-throughput GAG array

A SAHC30M sensor chip (streptavidin (SA) coupled to a 30 nm polycarboxylate matrix, Carterra 4294) was docked in a Carterra LSA^XT^, and the system primed in PBS with 0.05% Tween 20, pH 7.4 (PBST) buffer. The interaction temperature was set to 25 °C and the sample decks to 20 °C. The SA surface was conditioned with a 60s injection of 40 mM NaOH, then 16 different biotinylated GAGs were captured (10 min) to distinct spots on the chip surface to form a 96-ligand array. Each of the 16 GAGs were captured at one of six dilutions as a 5-fold dilution series, starting at approximately 0.5 μg/ml in the case of GAGs DP4 and DP8 (Iduron), and 8 μg/ml in the case of the other GAGs. Up to 160 response units (RU) of GAG was captured at the highest concentration. The development and optimization of the GAG array is described here (70). A 3-fold dilution series of both proteins was prepared in the same PBST buffer, using the range 3.5 nM to 7.62 μM in the case of four CHRD sample and 0.23 nM to 500 nM in the case of the shuttling complex with BMP2/7. Prior to the protein injections, eight buffer injections were performed to stabilise the baseline following GAG capture. Starting with the most dilute sample, increasing concentrations of protein were injected over the GAGs for 5 min, followed by 15 min of dissociation without surface regeneration between each protein injection. The data were processed by reference against the most adjacent streptavidin spot, then double-referenced using the buffer injection preceding the first protein injection. Following the alignment and appropriate cropping of the data, rate constants were obtained by fitting to the Langmuir 1:1 model. For the four CHRD regions, the data were typically fitted using 10 nM to 2.5 μM and for the Shuttling complex, data fitted using 2.1 nM to 167 nM using the first 5 min of dissociation.

### Biolayer interferometry (BLI)

All BLI measurements were performed on an Octet Red96 instrument (Sartorius Stedim) in an assay buffer of HBS pH 7.4 with 0.05% Tween-20 and 0.1 mg/ml BSA. DP8 and DP20 were biotinylated at their reducing end according to a previously published protocol (71). Biotinylated DP8, at a concentration of 0.5 μg/ml, and DP20, at a concentration of 5 μg/ml, were then captured onto Streptavidin-coated BLI sensors to a wavelength shift of 0.05 nm and 0.12 nm, respectively. Surfaces were then quenched with 100 μM biocytin for 120 s. Further non-immobilised sensors were quenched for use as reference sensors. Immobilised sensors and reference sensors were dipped into a concentration series of protein (Fig. S8). Experiments were performed in triplicate and new sensor surfaces were prepared following each concentration series. All sensors were double reference subtracted for analysis using the Octet data analysis HT v10 software. Fitting models used are as stated in the results section.

## Data availability

The structures of the four CHRD region and BioSAXS data generated in this study have been deposited in the PDB and SASBDB (72) databanks under accession codes: RCSB 9IGM (heparin oligomer-bound), 9RD6 (TEW-bound), 9QVV (R193A/R239A/R530A/H566A mutant), and SASDY96 (SAXS of the four CHRD region). Raw X-ray diffraction images are available at Zenodo with DOIs 10.5281/zenodo.19456138 for the heparin-bound form, 10.5281/zenodo.19456402 for the TEW-bound form, and 10.5281/zenodo.19456697 for the mutant form.

## Conflict of interest

The authors declare that they have no conflicts of interest with the contents of this article.
