# Supplementary material for: Structural and biophysical analysis of the four CHRD domains of human chordin reveals a novel binding site for glycosaminoglycans
Source: J Biol Chem. 2026 Jun 12;302(8):113248. doi: 10.1016/j.jbc.2026.113248 (PMC13382577; doi:10.1016/j.jbc.2026.113248)
Supplement: Supporting Figures and Tables [file mmc1.pdf]

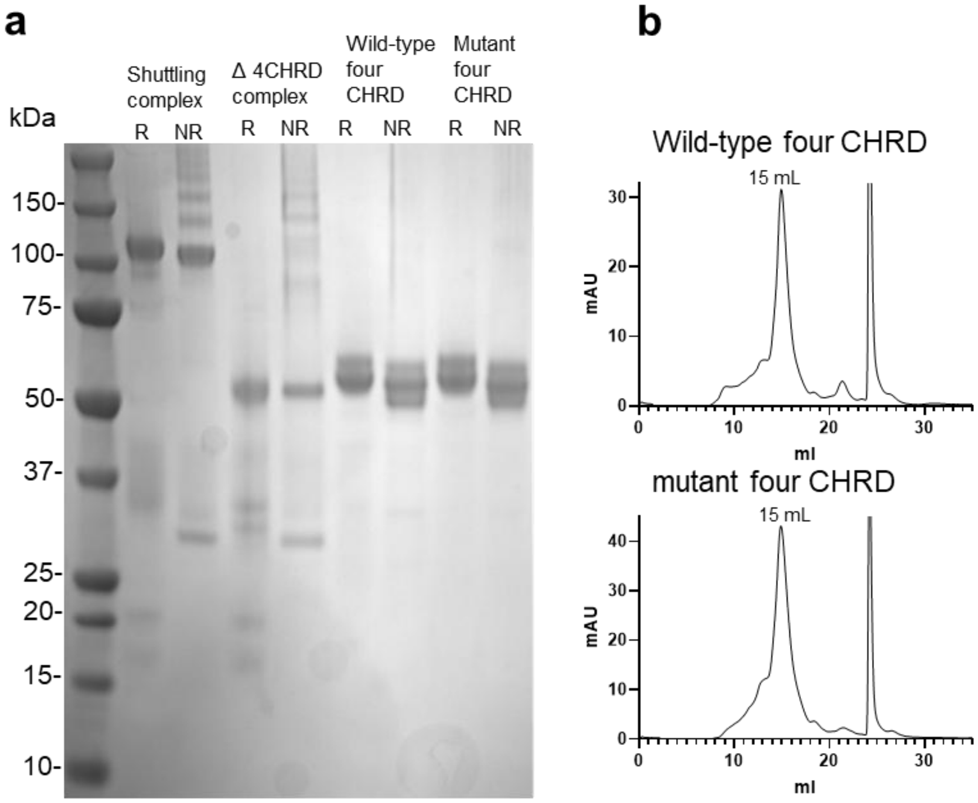

Supplementary figure 1: Purification of complexes and four CHRD domain variants

a) SDS PAGE analysis of purified complexes and four CHRD domains run under reducing (R) and non-reducing (NR) conditions, all lanes contained 10µL of protein at 0.16 mg/mL. Mutant refers to the four CHRD region containing R193A/R239A/R530A/H566A mutations. b) Size exclusion chromatographs showing the elution of the four CHRD domain variants at 15 mL from a Superdex 200 increase 10/300 GL column. Samples for crystallisation and biophysical analysis were taken from the centre of the main peak.

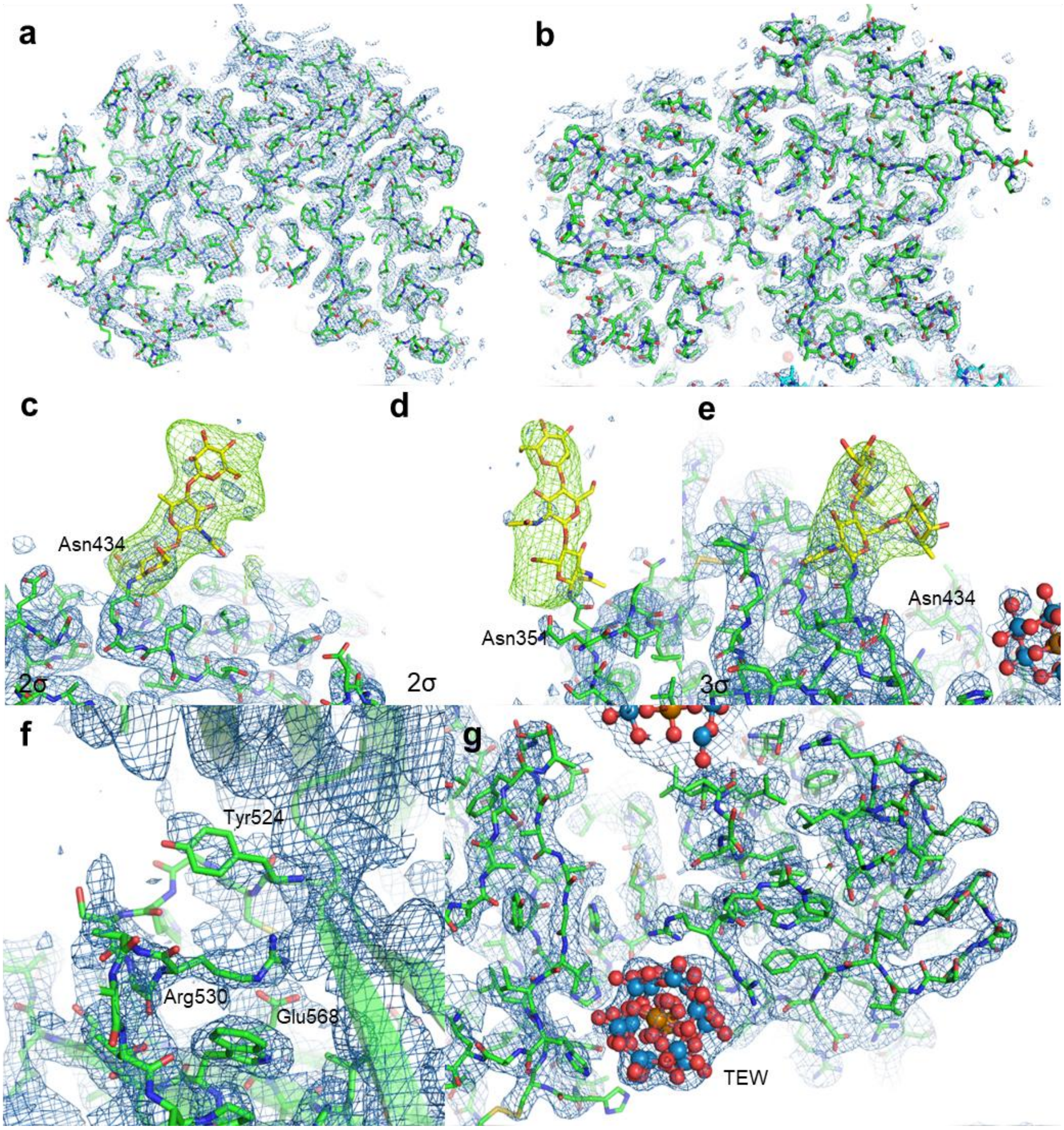

Supplementary figure 2: Electron density and model quality in X-ray crystal structures of the four CHRD domains

a) Electron density for most features from the heparin-bound structure (9IGM) is of reasonable quality. b) Despite the lower data resolution, the TEW-bound structure (9RD6) has sharper density in some regions and the ability to utilise the anomalous signal from the tungsten/tellurium clusters for MR-SAD and MLHL target function in PHENIX.refine makes these data more reliable for modelling. c) Diffuse density for an N-linked glycan is visible at Asn434 (9IGM). 2FO-FC density at 1 sigma is shown in blue and OMIT density at 2 sigma for the glycan is shown in green. d) Diffuse density for an N-linked glycan is visible at Asn351 (9IGM). 2FO-FC density at 1 sigma is shown in blue and OMIT density at 2 sigma for the glycan is shown in green. e) Different substituents of the glycan tree are visible between the four CHRD structures. In this example the core fucose moiety is visible at the 434 position in (9RD6). 2FO-FC density at 1 sigma is shown in blue and OMIT density at 3 sigma for the glycan is shown in green. f) The linker between CHRD domain 3 and CHRD domain 4 (9IGM) is poorly resolved and is clearly mobile. However, the atomic positions are constrained by the observable interaction between Arg530 and Glu568. g) The crystallisation adjuvant Tellurium-centred Anderson–Evans Polyoxotungstate interacts strongly with basic amino acids and binds in the glycosaminoglycan binding site (9RD6).

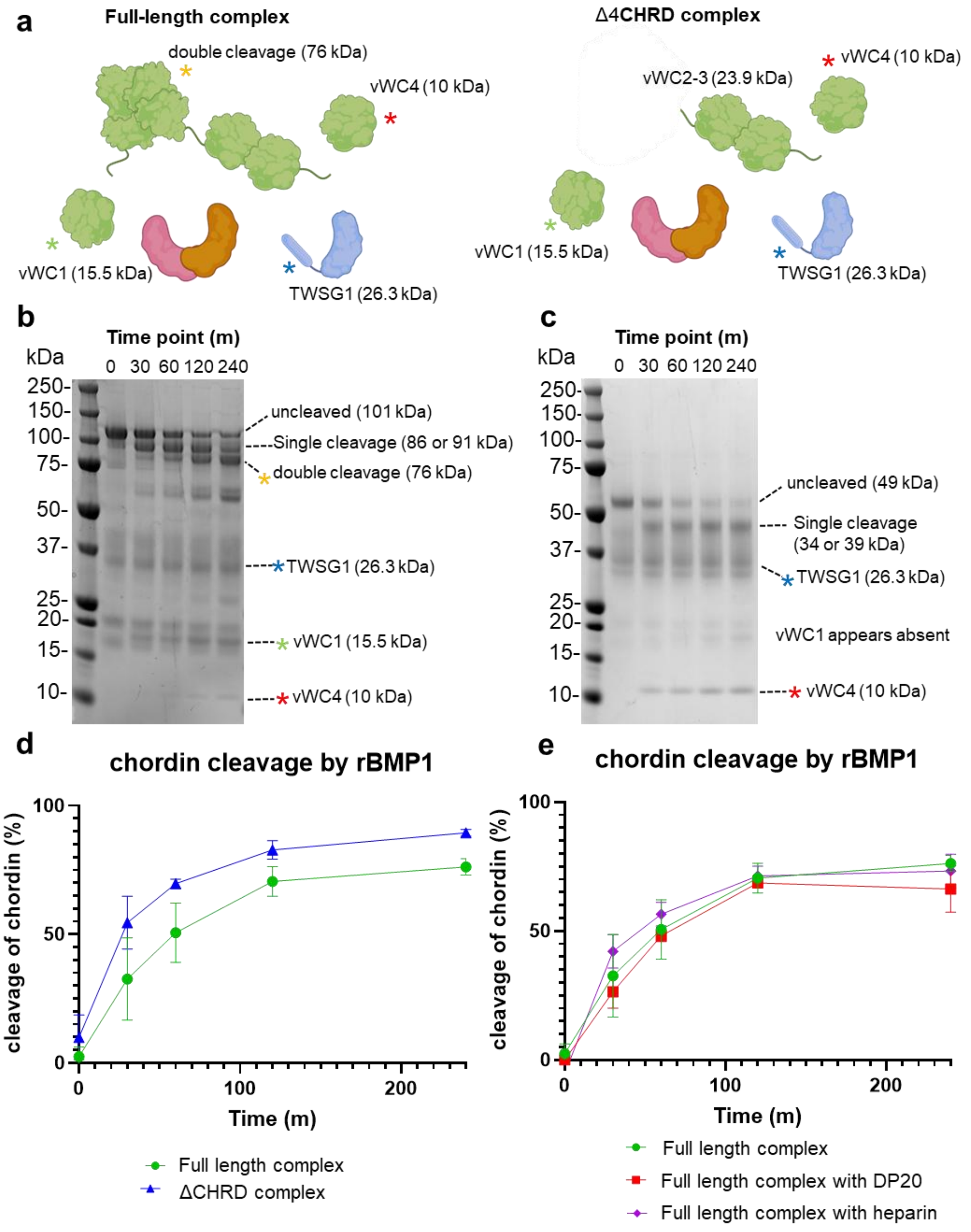

Supplementary figure 3: Cleavage of chordin complexes by rBMP1. a) Schematic showing the fragments expected upon cleavage of ternary complexes containing full length (left) and Δ4CHRD (right) variants of chordin. b) Representative SDS-PAGE from a BMP1 cleavage assay of the ternary complex of full-length chordin. The putative vWC1 fragment is shown with a green star, the putative vWC4 fragment is shown with a red star. TWSG1 is shown with a blue star. c) Representative SDS-PAGE from a BMP1 cleavage assay of the ternary complex of Δ4CHRD. The putative vWC4 fragment is shown with a red star, but the putative vWC1 fragment is absent. TWSG1 is shown with a blue star. These assays were repeated at least 3 times. d) Gel densitometry analysis of cleavage assays for full-length chordin (green) and Δ4CHRD (blue). e) Gel densitometry analysis of cleavage assays for full-length chordin in the absence (green) or presence of heparin oligosaccharide DP20 (red) and undigested heparin (purple). For (d) and (e), values plotted are technical repeats (N=3 for all reactions aside from full-length untreated (green) which features N=5). Full length untreated data features in both panels d and e.

Cleavage of Chordin Shuttling complexes

Gel densitometry analysis of the cleavage reactions appears to suggest that the Δ4CHRD samples undergo their first cleavage event more rapidly than full length chordin (Supplementary figure 3d). However, the two curves appear vertically displaced from one another and it appears that the cleavage of full-length chordin plateaus at a lower value, so it may be that a sub-population within the full-length complex is not cleaved, or the band intensity estimation is not comparable between the samples which were normalised by molarity (Supplementary figure 3d). It also appears that the Δ4CHRD samples can only be cleaved once, as there is a notable absence of a band for vWC1 which appears between the two BMP bands (Supplementary figure 3b,c, Supplementary figure 4) and the expected ~24 kDa species that would result from double cleavage (Supplementary figure 3a, c). The smallest cleavage product observed after digestion is consistent with the size of the vWC4 domain (~10 kDa) generated by cleavage at site 2 (MQA/DGPR), rather than the vWC1 fragment (~15.5 kDa), which would be produced by cleavage at site 1 (SYS/DRGE). Indeed, western blotting of the full-length chordin digestion demonstrates that the ~15.5 kDa fragment contains the N-terminal FLAG tag, confirming its identity as vWC1 (Supplementary figure 4). vWC4 appears earlier in the time course when the Δ4CHRD sample is digested, suggesting that the rate of cleavage may be faster. Both consensus cleavage sites are conserved in the Δ4CHRD construct with 14 residues of native peptide sequence retained between the cleavage site and the truncation site, but it appears that cleavage at the N-terminal site may be affected by deletion of the four CHRD domains, and C-terminal cleavage is enhanced. This may suggest that BMP1 interacts with the four CHRD domains and this interaction is a determinant of efficient cleavage at the upstream site. If the interaction between BMP1 and the four CHRD region is stronger than its interaction with the C-terminal region, then the full-length reactions might be expected to produce more product inhibition thus explaining the change in cleavage rate. As referenced in the main text, heparin does not appear to significantly alter the rate of cleavage of the full-length shuttling complex.

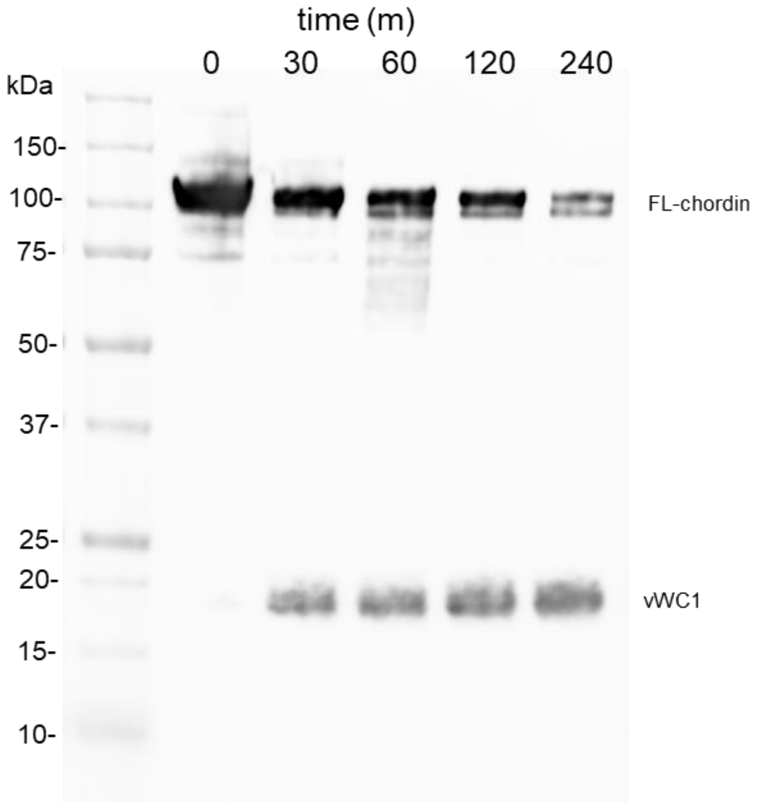

Supplementary figure 4. **Western blot of the rBMP1 digestion of the full-length shuttling complex**. Western blot detected with anti-FLAG antibody showing full-length chordin and the putative 15.5 kDa vWC1 domain fragment containing the N-terminal FLAG-tag. This fragment appears absent in the Coomassie stained gels of the Δ4CHRD digestions (Supplementary figure 3c).

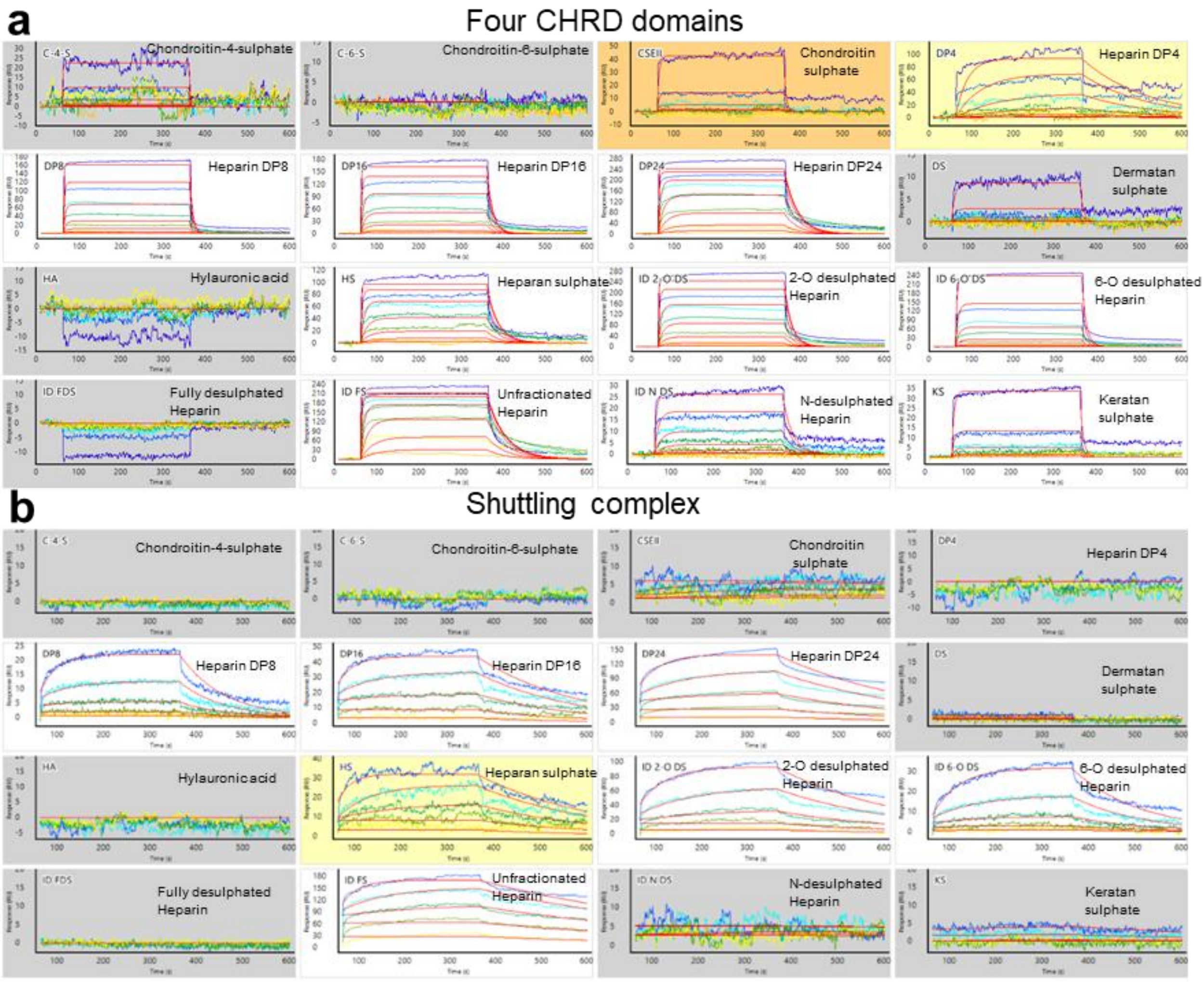

Supplementary figure 5: Example SPR binding data for four CHRD region and shuttling complexes to the full GAG array. a) SPR binding for the four CHRD domains. Tiles coloured grey denote no binding, tiles coloured yellow denote the standard deviation of the residuals is greater than 8% of the fitted R_{max}. Orange coloured tiles denote evidence of binding, but at too low affinity to quantify at the concentrations injected. The protein was added 10.5 nM to 7.6 uM as a 3-fold dilution series and the fit to the 1:1 model shown as a red line. b) SPR binding for the chordin-TWSG1-BMP2/BMP7 shuttle complex. Tiles coloured grey denote no binding, tiles coloured yellow denote the standard deviation of the residuals is greater than 5% of the fitted R_{max} The protein was added 0.69 to 167 nM as a 3-fold dilution series and the fit to the 1:1 model shown as a red line.

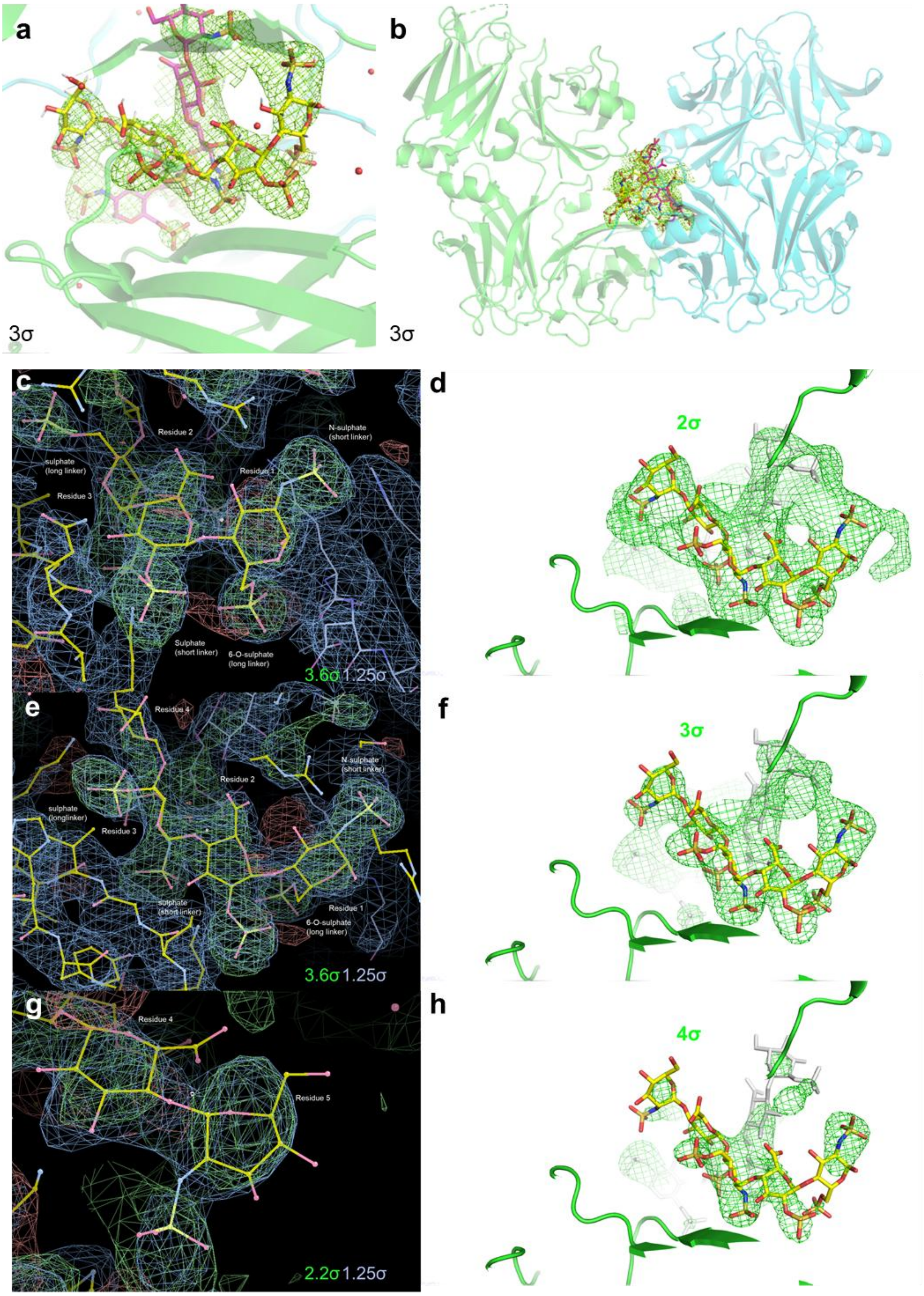

Supplementary figure 6: Heparin binding in the four CHRD region crystal structure

a) OMIT density for the heparin oligomer resolved in the crystal structure at 3 sigma. b) The putative binding sites face one another in the crystal lattice, and therefore the precise pose of the oligomer is distorted by interactions with multiple four CHRD assemblies. c,h) OMIT density at varying sigma levels shown in COOT (c, e, g), and Pymol (d, f h). sigma levels for Fo-Fc and 2Fo-fc are shown in green and blue respectively.

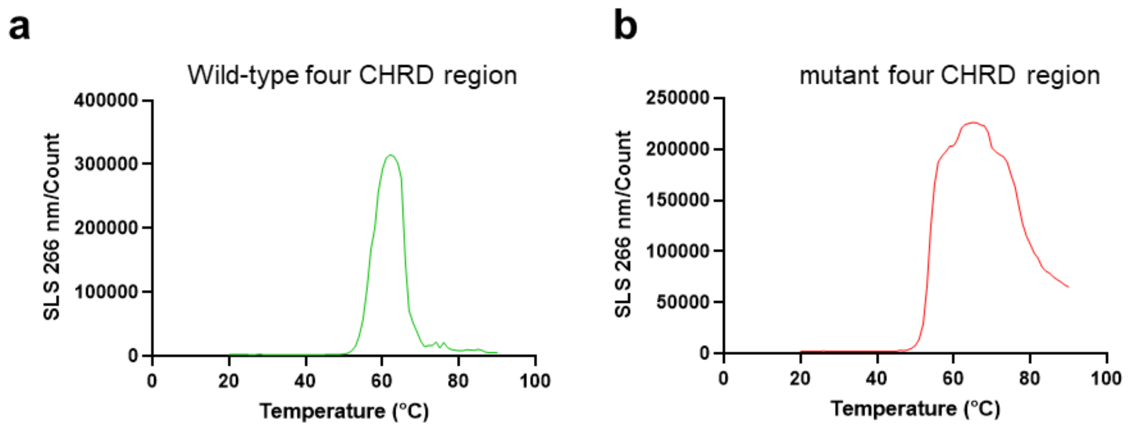

Supplementary figure 7. Static Light Scattering data at 266 nm for wildtype and mutant four CHRD region.

SLS data recorded at a range of temperatures from 20 – 90 ^{o}C. (a) Analysis of wild-type four CHRD region. b) Analysis of the R193A/R239A/R530A/H566A mutant four CHRD region.

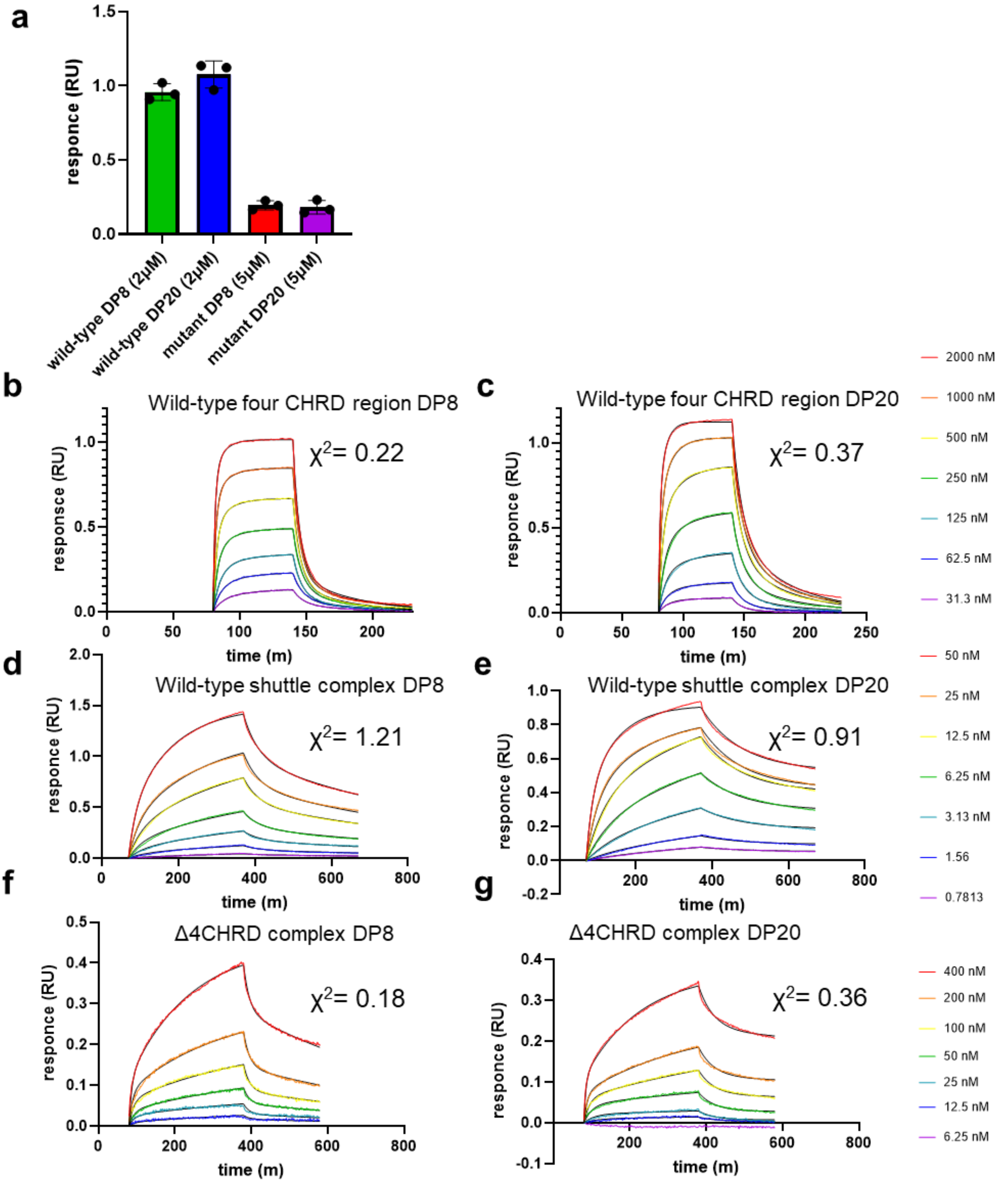

Supplementary figure 8: Octet-BLI interaction analysis of chordin samples to DP8 and DP20.

a) Octet BLI binding responses of wild-type and R193A/R239A/R530A/H566A mutant four CHRD region (analytes) to immobilised heparin DP8 and DP20. Data points are technical replicates (triplicate), error bars from standard deviation. b-g) Octet BLI traces for the four CHRD region, full-length shuttling complex and Δ4CHRD shuttling complex as analytes interacting with immobilised heparin oligosaccharides DP8 and DP20. Analyte concentrations are indicated and were the same for panels b and c; d and e; and f and g. Average χ2 values are shown and K_D values are stated in manuscript Figure 6.

X-ray crystal structures	4CHRD with heparin sulphate oligomer (9IGM)	4CHRD with anderson evans polyoxotungstate (9RD6)	4CHRD GAG binding mutant (9QVV)
**Data collection**			
Space group	P 62 2 2	P 43 2 2	I 2 2 2
Cell dimensions			
a, b, c (Å)	172.77, 172.77, 129.41	91.30, 91.30, 323.66	120.14, 123.80, 123.79
α, β, γ (°)	90.00, 90.00, 120.00	90.00, 90.00, 90.00	90.00, 90.00, 90.00
Resolution (Å)	2.92	3.28	2.72
Total unique reflections	25297 (3999)	44114 (8840)	24327 (2912)
I/σI	17.7 (1.0)	9.3 (1.1)	13.8 (1.0)
CC half	1.00 (0.530)	1.00 (0.522)	1.0 (0.768)
Completeness (%)	100 (100)	100 (100)	96.7 (89.2)
Multiplicity	78.9 (81.3)	48.6 (38.9)	13.5
Wilson B factor (Å2)	101.6	124.45	80.21
**Refinement**			
R/Rfree	0.231/0.273	0.210/0.247	0.2374/0.2794
R.M.S deviations			
Bond lengths (Å)	0.003	0.002	0.03
Bond angles (°)	0.629	0.619	0.616
Ramachandran outliers (%)	0	0	0
Molprobity score	2.33	1.86	2.03

Supplementary table 1: Crystallographic data and model quality statistics

Values for outer shells are shown in brackets

**bio-SAXS**	Chordin fragment aa.168–650 (SASDY96)
**Data collection**	
**Session ID**	mx31850-56
Beam diameter [μm]	1,000 × 250
Wavelength [Å]	0.9464
Energy [keV]	13.1
Flux [photons s−1]	4×10^{12}
Detector	Dectris EigerX 4M
Detector distance [m]	3.6883
q range [Å−1]	0.0045 – 0.34
Collection mode	In-line SEC-SAXS
SEC column	Superdex 200 increase 3.2/300
Concentration [mg ml−1]	9
Flow rate [ml min−1]	0.075
Exposure temperature [K]	288.2
Exposure time per frame [s]	1
Intensity scaling	Scaled to H2O (0.0163 cm−1)
**Data processing**	
Data reduction	DAWN
Data processing	ScÅtter IV
qRg range	0.2876 – 1.3260
Rg [Å]	29.62 ± 0.2
qmaxRg	1.326
**P(r) analysis**	
Dmax [Å]	105.7
Mr [kDa]	61.3
**Modelling**	
Ab initio model	
Software	DENSS
Model $\chi2$	1.24
Structure model	MultiFoXS
Input PDB	9RD6 (chain A)
Modelling	1 linker (1-state) / 2 linker (2-state)
Model $\chi2$	2.28 / 2.05

Supplementary Table 2: Small angle X-ray scattering data quality, ab initio density and MultiFoXS model quality statistics.
